# 人核因子-κBp65 shRNA慢病毒载体构建及对肺癌细胞恶性生物学行为的影响

**DOI:** 10.3779/j.issn.1009-3419.2014.05.02

**Published:** 2014-05-20

**Authors:** 红娟 郭, 光发 朱, 春婷 吴

**Affiliations:** 100029 北京，首都医科大学附属北京安贞医院呼吸与危重症医学科，北京市心肺血管疾病研究所 Department of Respiratory and Critical Care Medicine, Beijing Anzhen Hospital, Capital Medical University; Beijing Institute of Heart, Lung and Blood Vessel Diseases, Beijing 100029, China

**Keywords:** 核因子-κBp65, IκBα, 慢病毒载体, 稳转细胞株, 迁移, 粘附, NF-κBp65, IκBα, Lentiviral vector, Stable transfection cell line, Migration, Adhesion

## Abstract

**背景与目的:**

核因子-κB作为重要转录因子，与多种恶性肿瘤的发生发展有着密切联系，直接或间接抑制NF-κBp65的表达可逆转肿瘤细胞生物学行为。构建人核因子-κBp65基因shRNA重组质粒，感染A549细胞并筛选出稳定细胞株，对其迁移、粘附能力进行鉴定。

**方法:**

设计并合成人核因子-κBp65的乱序对照序列（scramble）和干扰序列（shRNA）构建重组质粒，在5’端引入一个*Bam*HI位点，3’端引入一个*Xho*I位点和*Eco*RI位点；感染A549细胞并由嘌呤霉素做稳转株的筛选；应用Western blot、qRT-PCR技术检测NF-κBp65的干扰效果及IκBα蛋白表达水平的变化；Transwell、MTT法分析其对A549细胞迁移、粘附能力的影响。

**结果:**

成功构建重组质粒并筛选出A549/NF-κB p65 scramble稳转株和A549/NF-κB p65 shRNA稳转株；A549/NF-κB p65 shRNA稳转细胞株与A549/NF-κB p65 scramble稳转细胞株及A549细胞相比NF-κBp65的mRNA、蛋白表达水平均下调；IκBα蛋白水平明显下调；细胞迁移能力及粘附能力均降低。

**结论:**

本实验通过RNA干扰技术构建的重组慢病毒可有效抑制NF-κBp65 mRNA和蛋白的表达水平；抑制NF-κBp65可明显降低A549细胞的迁移和粘附能力。

## Abstract (en)

**Background and objective:**

Nuclear factor-κB is an important transcription factor and is closely associated with a variety of malignant tumors. The biological behavior of lung tumor cells can be reversed by inhibiting the expression of NF-κBp65 directly or indirectly. Nuclear factor-κBp65 gene shRNA recombinant plasmids were constructed and then infected with A549 cells. New stable cell lines were selected, and the ability of migration and adhesion was identified.

**Methods:**

Both scramble control sequence and interference sequence (shRNA) of human nuclear factor-κBp65 were designed and synthesized to build recombinant plasmids, with *Bam*H I site at the 5′ end and *Xho* I and *EcoR* I sites at the 3′ end. A549 cells were infected, and stable transfection strains were selected by puromycin. Western blot and qRT-PCR methods were applied to assess the interference efficient of NF-κBp65 and the protein expression level of IκBα. Transwell and MTT assays were carried out to analyze the ability of migration and adhesion of A549 cells separately.

**Results:**

Recombinant plasmids were successfully built, and A549/NF-κB p65 scramble and A549/NF-κB p65 shRNA stable transfection strains were also successfully screened. Both mRNA and protein expression levels of NF-κBp65 showed that A549/NF-κBp65 shRNA cells decreased compared with A549/NF-κB p65 scramble cells and A549 cells, whereas the protein level of IκBα significantly increased. Both migration and adhesion abilities were also reduced.

**Conclusion:**

In this study, both mRNA and protein expression levels of NF-κBp65 were effectively suppressed by RNA interference technique. NF-κBp65 inhibition can significantly reduce the migration and adhesion ability of A549 cells.

每年肺癌的全球发病率超过150万例，中国的发病率尤其高；在中国的大多数城市，肺癌的发病率在各种癌症发病率中居首位；随着工业化的发展，环境污染的持续存在，据估计，肺癌的发病率和死亡率仍会持续上升^[1]^。核因子-κB（nuclear factor-κB, NF-κB）作为重要的核转录因子，与多种恶性肿瘤的发生发展有着密切联系；NF-κB通过与不同细胞因子的相互作用参与肿瘤细胞生长、增殖、侵袭等过程。通过对IKK激活、IκBα降解、IκBα核迁移及NF-κB与DNA结合等过程的抑制可直接抑制NF-κB的活化，从而不仅参与对细胞增殖、血管发生、肿瘤浸润和转移的调节，亦可增强癌细胞对化疗药物的敏感性^[2]^。近年来，有关动物模型和细胞培养的研究已经确定了NF-κB和肺癌发生之间的联系，并且强调了针对NF-κB信号通路对肺癌进行治疗和化学预防的意义^[3]^。NF-κB蛋白家族有p65（RelA）、RelB、c-Rel、p50/p105（NF-κB 1）和p52/p100（NF-κB 2）五个亚基，RelA（p65）是NF-κB活化途径的一个关键组成部分，具有广泛的调节作用，不仅参与细胞增殖及抗细胞凋亡的调节，而且在组织浸润、迁移、转移等肿瘤生物学行为中也发挥一定的作用^[4, 5]^。本研究利用RNA干扰技术构建人NF-κBp65短发卡RNA（short hairpin RNA, shRNA）慢病毒载体，转染293T细胞包装慢病毒，感染A549细胞并筛选出稳转株，检测对NF-κBp65的干扰效果、对抑制性κBα（inhibitor-κBα, IκBα）表达水平的影响及对A549细胞迁移、粘附能力的影响，进一步研究NF-κBp65和IκBα的关系及两者对肺癌细胞生物学行为的影响，探讨NF-κBp65、IκBα在癌症预防和治疗中的作用。

## 1 材料与方法

### 1.1 材料

pLVX-shRNA-tdTomato-puro（红色荧光）慢病毒干扰载体质粒及慢病毒包装细胞293T细胞（深圳百恩维生物公司）；肺腺癌细胞系A549细胞（购于中国协和医科大学基础研究所细胞中心）；大规模质粒抽提试剂盒（德国Qiagen公司），DNA Marker（北京全式金生物公司），限制性内切酶*Eco*RI、*Bam*HI、*Xho*I和T4 DNA连接酶（美国NEB公司），胎牛血清（fetal bovine serum, FBS）、DMEM培养基、0.25%胰酶和Trizol试剂（美国Invitrogen公司），高效转染试剂盒（深圳百恩维生物公司提供），AMV反转录试剂盒（美国Promega公司），SYBR Green（Takara公司）。鼠抗人NF-κBp65单克隆抗体、鼠抗人IκBα单克隆抗体及鼠抗人GAPDH一抗（美国Cell Signal公司），24孔transwell小室（孔径8 μm）（Corning Costar公司）。

### 1.2 方法

#### 1.2.1 人NF-κBp65 shRNA慢病毒载体构建

根据GenBank中人NF-κBp65基因序列^[6]^，从invitrogen公司合成shRNA片段，其中在5’端引入一个*Bam*HI位点，3’端引入一个*Xho*I位点和一个*Eco*RI位点，所设计乱序对照序列（scramble）和干扰序列（shRNA）见表 1。pLVX-shRNA-tdTomato-Puro载体质粒由限制性内切酶*Bam*HI和*Eco*RI酶进行双酶切后，分别与NF-κBp65 scramble和NF-κBp65 shRNA通过T4 DNA连接酶连接，转化至JM109感受态细胞中，挑取单菌落，提取质粒，进行酶切电泳及基因测序。

**1:** 基因序列 Gene sequences

Name	Sequence
Scramble control RNA sequence	scramble-F：
	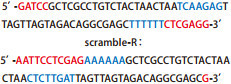
Interference RNA sequence	NF-*κ*Bp65-F：
	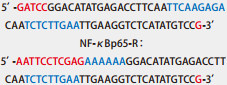
Red: restriction enzyme sites; blue: loop rings and A tails; black: target sequences.	

#### 1.2.2 慢病毒包装及病毒滴度测定

在10 cm培养皿接种（6-8）×10^6^ 293T细胞，37 ℃，5%CO_2_培养箱中培养；待细胞密度达70%-80%时开始转染，转染前2 h-4 h更换培养基为DMEM+10%FBS培养基；按慢病毒包装试剂盒说明配制转染液，混匀后室温静置30 min，逐滴均匀加入培养皿中，然后置于培养箱中培养；12 h-16 h后改换为新鲜培养基，分别于24 h和48 h后收集上清；两次上清混合后1, 000 rpm离心5 min，0.45 μm PVDF滤器过滤至50 mL离心管中，4 ℃，50, 000 g高速离心2 h，弃上清，PBS重悬病毒沉淀，室温静置2 h，用移液器轻轻地吹匀，室温静置30 min，分装，-80 ℃冰箱保存。

慢病毒滴度测定方法：于测定前一天取48孔板，按3×10^4^/孔接种HEK293细胞，并在完全培养基中[DMEM+10%FBS+青/链霉素（P/S）]加入多聚季丁胺，使其终浓度为8 μg/mL；用上述培养基10倍梯度稀释病毒，然后分别吸取10 μL病毒稀释液到相对应的48孔板中，37 ℃，5%CO_2_培养箱中培养过夜，16 h-24 h后换液；继续培养48 h后，荧光显微镜计数荧光细胞数量。

#### 1.2.3 A549稳转细胞株的筛选

在6孔板中按6×10^5^/孔接种A549细胞，完全培养基培养；次日，换为含有8 μg/mL多聚季丁胺的完全培养基并以感染复数（multiplicity of infection, MOI）=10加入病毒；第3日换为完全培养基培养，48 h后消化并传代；待细胞贴壁后按5 μg/mL的浓度加入嘌呤霉素做筛选，并每2 d-3 d换液进行稳转株的筛选；一星期后换为含浓度为2 μg/mL嘌呤霉素的完全培养基；等细胞长满后消化、扩大培养。

#### 1.2.4 干扰效果的鉴定

##### 1.2.4.1 实时荧光定量-PCR（real-time quantitative polymerase chain reaction, qRT-PCR）

将A549细胞、A549/NF-κB p65 scramble和A549/NF-κB p65 shRNA稳转株分别接种在6孔板中，待细胞长满后加Trizol提取细胞总RNA，纯化mRNA，反转录获得cDNA。根据genbank中人NF-κBp65基因的mRNA序列设计引物，以人*GAPDH*基因作为内参；NF-κBp65 cDNA引物序列：上游5' -CCCCACGAGCTTGTAGGAAAG-3' ，下游5' -CCAGGTTCTGGAAACTGTGGAT-3' ，扩增片段为128 bp；GAPDH cDNA引物序列：上游5' -AGCCACATCGCTCAGACAC-3' ，下游5' -GCCCAATACGACCAAATCC-3' ，扩增片段为66 bp，均由北京诺赛基因组研究中心有限公司合成。20 μL反应体系，反应条件为：95 ℃ 5 min，进入45次循环（95 ℃ 45 sec，60 ℃ 1 min采集荧光），最后55 ℃ 5 min（绘制溶解曲线）。实验重复进行3次。qRT-PCR反应体系为20 μL体系，反应条件：50 ℃ 2 min，95 ℃ 10 min，95 ℃ 30 s，60 ℃ 30 s，40个循环。实验重复进行3次。

##### 1.2.4.2 Western blot

将A549细胞、A549/NF-κB p65 scramble和A549/NF-κB p65 shRNA稳转株分别接种在6孔板中，待细胞长满后加0.25%胰酶200 μL消化细胞，置于1.5 mL离心管中，离心弃上清后加入50 μL RIPA组织裂解液置于冰上，反复震荡、充分裂解，离心取上清。BCA法测蛋白浓度，将各组织样品浓度调至相同，保证每个电泳孔道加入60 μg蛋白混合样品，进行聚丙烯酰胺凝胶电泳、转膜。5%脱脂牛奶（TBST溶解）封闭，室温孵育60 min；加入1:1, 000稀释（1% BSA-PBS稀释）的NF-κBp65、IκBα一抗，1:800稀释的GAPDH 4 ℃过夜；次日PBS洗10 min×3次，加入1:3, 000稀释的抗NF-κBp65、抗IκBα荧光二抗及1:5, 000稀释的抗GAPDH荧光二抗，室温孵育1 h，PBS洗10 min×3次。使用化学发光成像系统显色拍照。以同一泳道中NF-κBp65和GAPDH条带灰度值之比反映NF-κBp65蛋白表达水平，IκBα和GAPDH条带灰度值之比反映IκBα蛋白表达水平。

#### 1.2.5 细胞迁移、粘附能力的鉴定

##### 1.2.5.1 细胞迁移-transwell

取对数生长期细胞，饥饿培养过夜，0.25%胰酶消化，调整细胞密度为5×10^5^/mL，取200 μL细胞悬液加至24孔transwell小室的上室，下室预先加入600 μL含有5%FBS的DMEM培养基，37 ℃、5%CO_2_培养箱培养12 h；取出小室，多聚甲醛固定10 min；PBS洗2遍，DAPI染核，显微镜下观察计数。

##### 1.2.5.2 细胞粘附实验——MTT法

取10 mg/mL BSA和10 mg/L纤维粘连蛋白按50 μL/孔加入96孔板中，4 ℃过夜，BSA为对照基底；次日吸出多余液体，每孔加入50 μL含10 mg/mL牛血清白蛋白（bovine serum albumin, BSA）的无血清培养基，37 ℃静置30 min；0.25%胰酶消化细胞，调整细胞浓度为1×10^5^/mL，各取100 μL细胞悬液接种于96孔板中，1 h后弃上清，PBS清洗2次-3次。溴化-3（4, 5-二甲基噻唑基-2）-2, 5-二苯基四唑[3-(4, 5-Dimethylthiazol-2-yl)-2, 5-diphenyltetrazolium bromide, MTT]法测定各孔吸光度（optical density, OD）值，以BSA组贴壁细胞OD值为参照，计算细胞粘附率。

粘附率计算公式：粘附率=[（实验组细胞OD值/对照组细胞OD值）-1]×100%。

### 1.3 统计学处理

本实验采用SPSS 17.0统计软件对实验结果进行统计分析，计量资料采用Mean±SD表示，多组样本间的比较采用单因素方差分析，如果有统计学差异，多个样本均数间的两两比较用*q*检验，计数资料用卡方检验，以α=0.05为检验水准，*P* < 0.05为差异有统计学意义。

## 2 结果

### 2.1 人NF-κBp65 shRNA慢病毒载体构建结果

将具有氨苄抗性的阳性克隆菌株小量快速提取质粒，分别用XhoI限制性内切酶进行酶切鉴定，1%琼脂糖凝胶电泳可见2, 065 bp的条带（图 1）。对所提质粒进行命名，分别命名为NF-κBp65 scramble病毒载体和NF-κBp65 shRNA病毒载体挑取酶切鉴定正确的菌液去测序，测序引物为U6-F：5’-TACGATACAAGGCTG-TTAGAGAG-3’，由深圳百恩维生物公司完成测序，其结果显示：NF-κBp65 scramble和NF-κBp65 shRNA的测序结果均与设计序列一致。证实NF-κBp65 scramble和NF-κBp65 shRNA慢病毒载体构建成功（图 2）。

**1:**
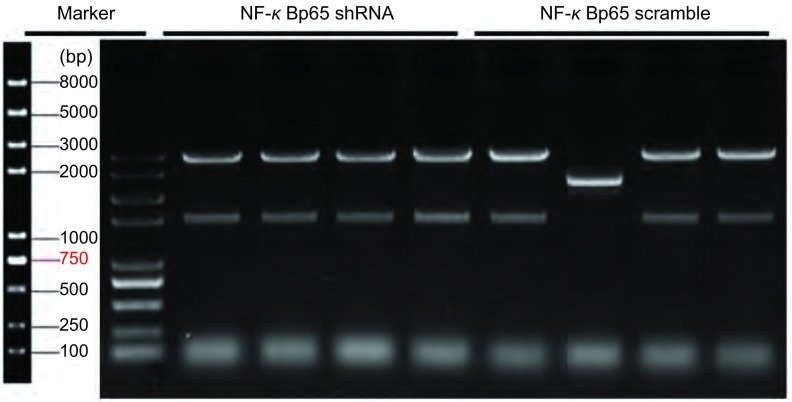
NF-*κ*Bp65 shRNA慢病毒载体和NF-*κ*Bp65 scramble慢病毒载体酶切鉴定结果 Identified results of NF-*κ*Bp65 shRNA lentiviral vector and NF-*κ*Bp65 scramble lentiviral vector

**2:**
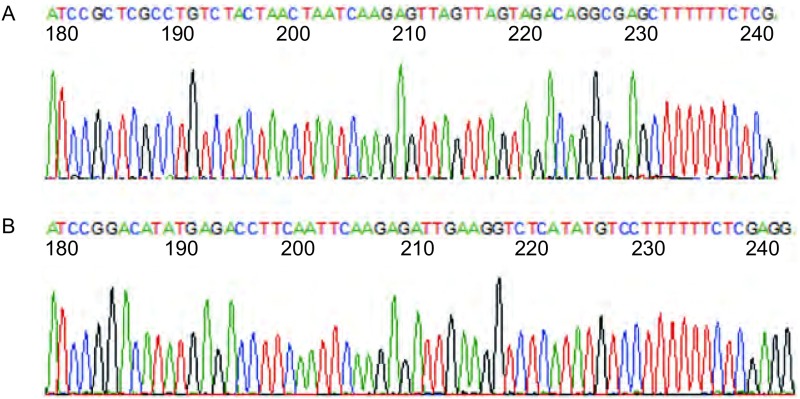
乱序对照序列和干扰序列测序结果（部分）。A: NF-*κ*Bp65 scramble测序结果；B: NF-*κ*Bp65 shRNA测序结果。 Sequencing results of scramble control sequences and interference sequences (partly). A: sequencing result of scramble control sequences; B: sequencing result of interference sequences.

### 2.2 慢病毒包装结果

pLVX-shRNA-tdTomato-Puro载体质粒带有红色荧光，NF-κBp65 scramble慢病毒载体和NF-κBp65 shRNA慢病毒载体经293T细胞包装后可在荧光显微镜下观察到发红色荧光的293T细胞（图 3）。

**3:**
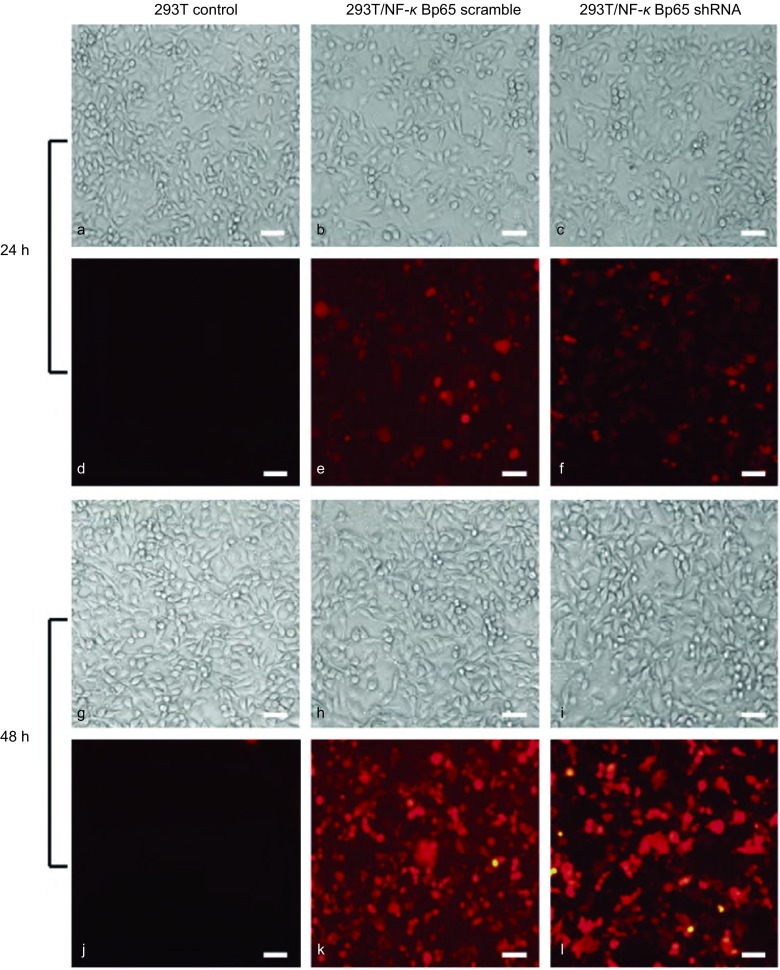
293T细胞对NF-*κ*Bp65 scramble和NF-*κ*Bp65 shRNA慢病毒载体的包装结果。a、b、c、d、e和f为转染24 h后293T细胞；g、h、i、j、k和l为转染48 h后293T细胞。其中a、b、c、g、h、i为自然光下293T细胞；d、e、f、j、k、l为红色荧光下293T细胞（bar=100 *μ*m）。 Packaging results of NF-*κ*Bp65 scramble lentiviral vector and NF-*κ*Bp65 shRNA lentiviral vector by 293T cells. a, b, c, d, e and f are 293T cells that have been transfected 24 h; g, h, i, j, k and l are 293T cells that have been transfected 48 h; a, b, c, g, h and i are 293T cells under white light; d, e, f, j, k and l are 293T cells under red fluorescence (bar=100 *μ*m).

### 2.3 A549稳转细胞株的筛选和鉴定结果

经过数日筛选出的稳转细胞株与未转染的A549细胞对比观察（图 4）。

**4:**
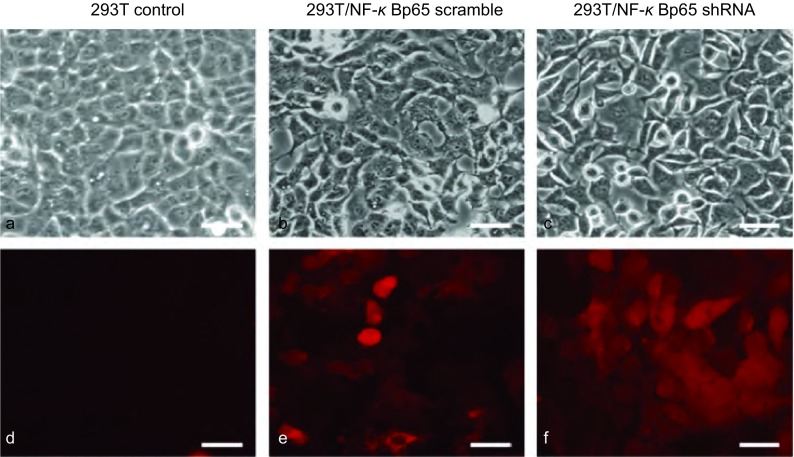
筛选出的A549/NF-*κ*Bp65 scramble稳转细胞株和A549/NF-*κ*Bp65 shRNA稳转细胞株。a、b和c为自然光下A549细胞；d、e和f为红色荧光下A549细胞（bar=50 *μ*m）。 Stably transfected cell strains of A549/NF-*κ*Bp65 scramble and stably transfected cell strains of A549/NF-*κ*Bp65 shRNA that have been screened out. a, b and c are A549 cells under white light; d, e and f are A549 cells under red fluorescence (bar=50 *μ*m).

### 2.4 NF-κBp65干扰效果的鉴定结果

#### 2.4.1 qRT-PCR结果

A549细胞与A549/NF-κBp65 scramble细胞间的NF-κBp65 mRNA表达水平（1.03±0.18 *vs* 0.97±0.10）无明显差异（*P*=0.802, 3），A549/NF-κBp65 shRNA细胞的NF-κBp65 mRNA表达水平（0.25±0.07）明显低于A549细胞和A549/NF-κBp65 scramble细胞（*P* < 0.05, *P* < 0.01），与A549细胞相比NF-κBp65 mRNA的表达水平下降75.3%（图 5）。

**5:**
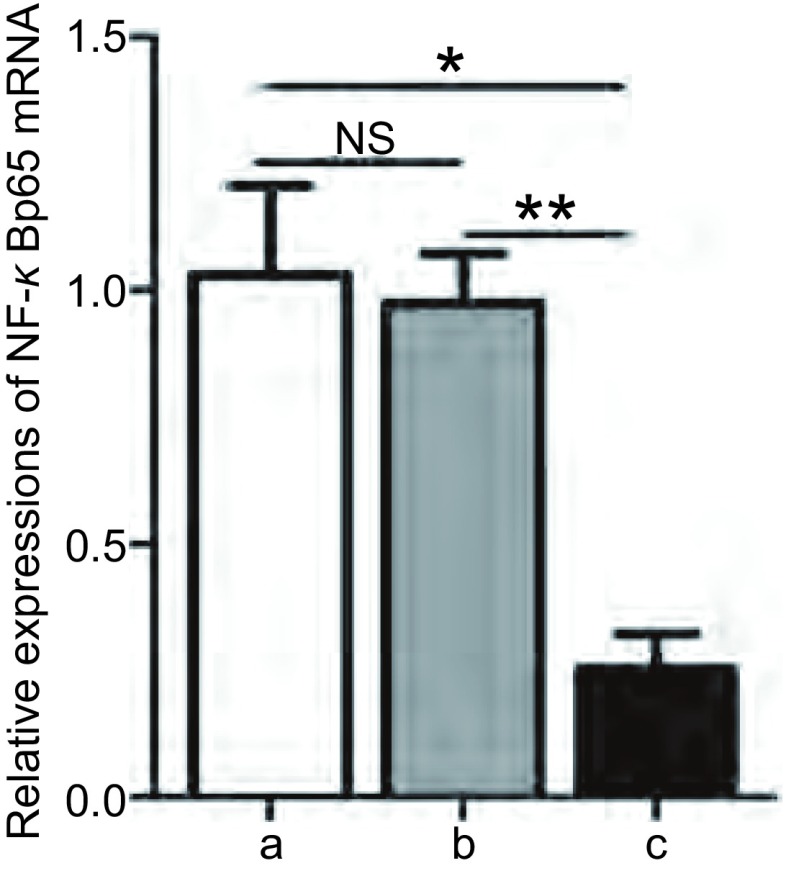
不同A549稳转细胞株中NF-*κ*Bp65的mRNA表达水平。转染NF-*κ*Bp65 shRNA后的A549细胞NF-*κ*Bp65 mRNA表达水平较对照组A549细胞和转染NF-*κ*Bp65 scramble后的A549细胞均明显降低；转染NF-*κ*Bp65 scramble后的A549细胞NF-*κ*Bp65 mRNA表达水平与对照组A549细胞无明显差异。a：A549细胞；b：A549/NF-*κ*Bp65 scramble细胞；c：A549/NF-*κ*Bp65 shRNA细胞。NS：*P* > 0.05；^*^P < 0.05；^**^P < 0.01。 Expression levels of NF-*κ*Bp65 mRNA in different stably transfected A549 cell strains Expression level of NF-*κ*Bp65 mRNA was significantly reduced in A549/NF-*κ*Bp65 shRNA cells than that in A549 cells and A549/NF-*κ*Bp65 scramble cells, but no difference between A549 cells and A549/NF-*κ*Bp65 scramble cells. a: A549 cell; b: A549/NF-*κ*Bp65 scramble cell; c: A549/NF-*κ*Bp65 shRNA cell; NS: *P* > 0.05; ^*^P < 0.05; ^**^P < 0.01.

#### 2.4.2 Western blot结果

A549细胞与A549/NF-κBp65 scramble细胞间NF-κBp65蛋白表达水平（0.57±0.07 *vs* 0.49±0.04, *P*=0.394, 9）无明显差异，A549/NF-κBp65 shRNA细胞的NF-κBp65蛋白表达水平（0.24±0.03）明显低于A549细胞和A549/NF-κBp65 scramble细胞（*P* < 0.05, *P* < 0.01），与A549细胞相比NF-κBp65蛋白表达水平下降52.8%；A549细胞与A549/NF-κBp65 scramble细胞间IκBα蛋白表达水平（0.43±0.04 *vs* 0.36±0.01, *P*=0.201, 5）无明显差异，A549/NF-κBp65 shRNA细胞的IκBα蛋白表达水平（0.22±0.02）明显低于A549细胞和A549/NF-κBp65 scramble细胞（*P*均 < 0.01）（图 6）。

**6:**
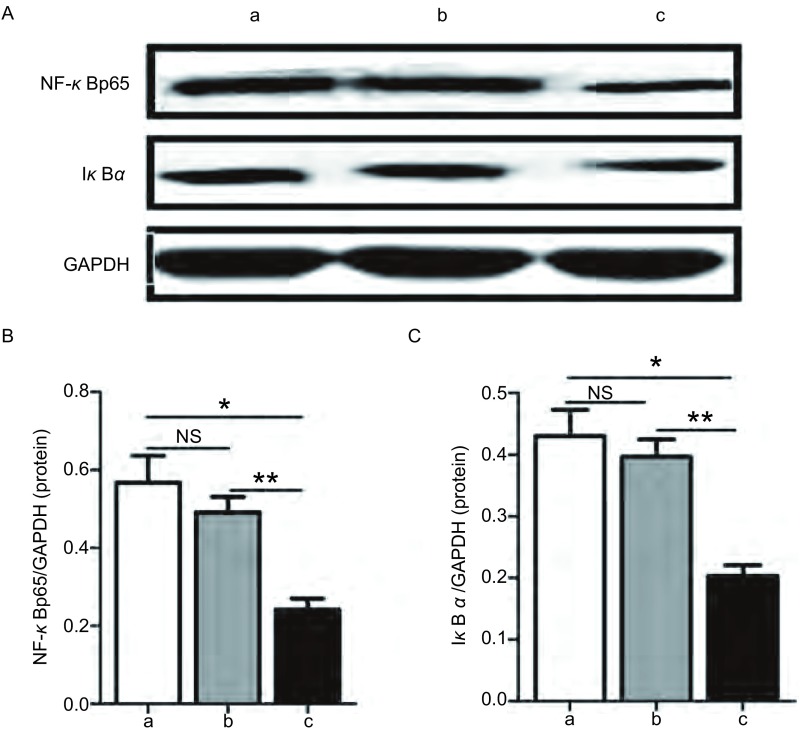
不同A549稳转细胞株中NF-*κ*Bp65蛋白和I*κ*B*α*蛋白表达水平。A: A549/NF-*κ*Bp65 shRNA细胞及对照组A549细胞和A549/NF-*κ*Bp65 scramble细胞中NF-*κ*Bp65蛋白和I*κ*B*α*蛋白的western blot结果，以GAPDH蛋白为内参；B:转染NF-*κ*Bp65 shRNA后的A549细胞NF-*κ*Bp65蛋白表达水平较对照组A549细胞和转染NF-*κ*Bp65 scramble后的A549细胞均明显降低；转染NF-*κ*Bp65 scramble后的A549细胞NF-*κ*Bp65蛋白表达水平与对照组A549细胞无明显差异；C:转染NF-*κ*Bp65 shRNA后的A549细胞I*κ*B*α*蛋白表达水平较对照组A549细胞和转染NF-*κ*Bp65 scramble后的A549细胞均明显降低；转染NF-*κ*Bp65 scramble后的A549细胞I*κ*B*α*蛋白表达水平与对照组A549细胞无明显差异。a：A549细胞；b：A549/NF-*κ*Bp65 scramble细胞；c：A549/NF-*κ*Bp65 shRNA细胞。NS：*P* > 0.05；*P < 0.05；**P < 0.01。 Expression levels of NF-*κ*Bp65 protein and I*κ*B*α* protein in different stably transfected A549 cell strains. A: The western blot results of NF-*κ*Bp65 protein and I*κ*B*α* protein in A549/NF-*κ*Bp65 shRNA cells, A549 cells and A549/NF-*κ*Bp65 scramble cells. GAPDH served as an internal control; B: Expression level of NF-*κ*Bp65 protein was significantly reduced in A549/NF-*κ*Bp65 shRNA cells than that in A549 cells and A549/NF-*κ*Bp65 scramble cells, but no difference between A549 cells and A549/NF-*κ*Bp65 scramble cells; C: Expression level of I*κ*B*α* protein was significantly reduced in A549/NF-*κ*Bp65 shRNA cells than that in A549 cells and A549/NF-*κ*Bp65 scramble cells, but no difference between A549 cells and A549/NF-*κ*Bp65 scramble cells. a: A549 cell; b: A549/NF-*κ*Bp65 scramble cell; c: A549/NF-*κ*Bp65 shRNA cell. NS: *P* > 0.05; *P < 0.05; **P < 0.01.

### 2.5 细胞迁移能力鉴定结果

A549细胞与A549/NF-κBp65 scramble细胞相比细胞迁移数无明显差异（290.4±14.7 *vs* 278.6±18.0, *P*=0.6258），A549/NF-κBp65 shRNA细胞的迁移数（118.2±13.9）明显少于A549细胞和A549/NF-κBp65 scramble细胞（*P*均 < 0.01）（图 7A-图 7D）。

**7:**
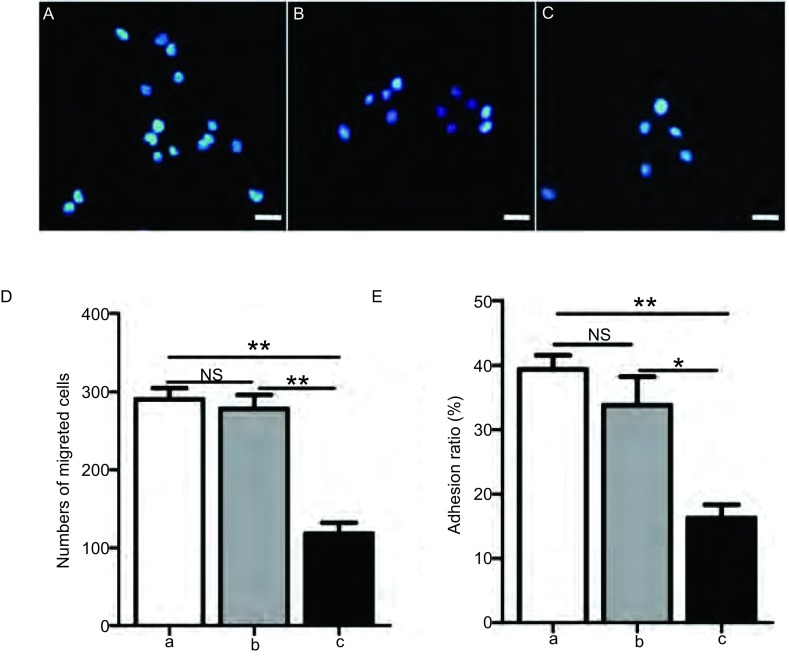
不同A549稳转细胞株间细胞迁移数、粘附率的比较。A、B、C:分别为A549细胞、A549/NF-*κ*Bp65 scramble细胞和A549/NF-*κ*Bp65 shRNA细胞经transwell板上小孔发生迁移细胞的代表性视野（200×, bar=100 *μ*m）；D: A549/NF-*κ*Bp65 shRNA细胞的迁移能力明显低于A549细胞和A549/NF-*κ*Bp65 scramble细胞；E: A549/NF-*κ*Bp65 shRNA细胞的粘附率明显低于A549细胞和A549/NF-*κ*Bp65 scramble细胞。a：A549细胞；b：A549/NF-*κ*Bp65 scramble细胞；c：A549/NF-*κ*Bp65 shRNA细胞。NS：*P* > 0.05；*P < 0.05；**P < 0.01。 Comparisons about numbers of migrated cells and adhesion ratio among different stably transfected A549 cell strains. A, B and C showed representative fields of cells migrated through filters in transwell inserts from A549, A549/NF-*κ*Bp65 scramble and A549/NF-*κ*Bp65 shRNA cells respectively (200×, bar=100 *μ*m); D: The ability of migration was significantly reduced in A549/NF-*κ*Bp65 shRNA cells than that in A549 cells and A549/NF-*κ*Bp65 scramble cells, but no difference between A549 cells and A549/NF-*κ*Bp65 scramble cells; E: The adhesion ratio of A549/NF-*κ*Bp65 shRNA cells was significantly reduced than that in A549 cells and A549/NF-*κ*Bp65 scramble cells, but no difference between A549 cells and A549/NF-*κ*Bp65 scramble cells. a: A549 cell; b: A549/NF-*κ*Bp65 scramble cell; c: A549/NF-*κ*Bp65 shRNA cell. NS: *P* > 0.05; *P < 0.05; **P < 0.01.

### 2.6 细胞粘附率鉴定结果

A549细胞、A549/NF-κBp65 scramble细胞、A549/NF-κBp65 shRNA细胞粘附率的均值分别为39.4%、33.8%、16.2%。A549细胞和A549/NF-κBp65 scramble细胞粘附率[（39.4±2.2）% *vs*（33.8±4.5）%]无明显差异（*P*=0.304, 8）；A549/NF-κBp65 shRNA细胞的粘附率（16.3±2.1）%明显低于A549细胞和A549/NF-κBp65 scramble细胞（*P* < 0.01, *P* < 0.05）（图 7E）。

## 3 讨论

NF-κB通过介导过度炎症反应，引起肺部反复的损伤和修复，细胞更新增快，基因突变累积，最终导致肺癌的发生^[7]^。近年来，越来越多的研究证明NF-κB是肿瘤发生、发展的重要促进因子。NF-κB可诱导参与细胞生长、粘附、分化、增殖等不同过程的200多个基因的表达^[3]^。通过参与细胞迁移和粘附过程，NF-κB可促进肿瘤细胞的转移^[5]^。

作为NF-κB蛋白家族的主要成员，Rel（p65）基因异常扩增或上游调节因子的异常活化可引起NF-κB活化失调，促进自身免疫性疾病、慢性炎症及癌症的发生^[8]^。IκB蛋白的表达是NF-κB活化的最敏感标志之一，通过检测IκBα磷酸化和降解可明确NF-κB是否发生活化，IκBα作为NF-κB的抑制物，其表达水平在NF-κB活化过程恢复后下调^[4, 9]^。对已活化的NF-κB有多种机制可使其表达水平下调，包括新合成的IκBα在内的特征性反馈环路；其中IκBα主要是通过与DNA“竞争性”结合核NF-κB，进而使NF-κB与DNA解离后进入细胞质^[9]^。

有研究显示，直接抑制NF-κB相关基因或间接干扰其相关转录激活因子均可发挥与转录抑制因子相同的功能，即介导NF-κB的活化。直接抑制包括作用NF-κB活化途径的不同成分或过程，例如IKK的激活，IκBα的降解，核转位及NF-κB与DNA绑定，其中IKK被认为是最有效的选择性药物靶点；间接抑制是指抑制可激活NF-κB活化途径的蛋白但并非是组成成分^[3]^。Kasperczyk等^[10]^的研究表明，IκBα的超级抑制物或p65基因敲除可特异性抑制NF-κB活化；Chen等^[4]^的研究表明：通过siRNA途径敲除IKKβ或RelA比超级抑制物IκBα能更好的阻断A549细胞中NF-κB的活化。

RNA干扰是一个高特异、高选择抑制目标基因表达的自然过程，在癌症的个体化治疗方面有重要价值，已被广泛应用于探索基因功能和传染性疾病及恶性肿瘤基因治疗等领域^[11, 12]^。

本研究结果发现：NF-κBp65受抑制后，其抑制因子IκBα的蛋白水平较空白对照组A549细胞和乱序对照组A549细胞均明显下调；静息状态下，p65/p50异源二聚体与IκBα在细胞质中以聚合物形式存在，大多数刺激因子通过IKK介导IκBα N-末端丝氨酸残基的磷酸化激活NF-κB^[13]^。这可能是因为干扰NF-κBp65基因后A549细胞中NF-κBp65蛋白水平下降，此时“多余”的IκBα无法与NF-κBp65/p50结合，发生了降解，较未受干扰的A549细胞中的IκBα表达量降低。

NF-κBp65基因被干扰后的A549细胞迁移能力及粘附率较空白对照组A549细胞和乱序对照组A549细胞均明显降低。NF-κB通过对MMPs、血管细胞粘附分子-1（vascular cell adhesion molecule-1, VCAM-1）、胞间粘附因子-1（intercellular adhesion molecule-1, ICAM-1）、趋化因子受体CXCR4和丝氨酸蛋白酶尿激酶型纤维蛋白酶原激活剂（serine protease urokinase-type plasminogen activator, uPA）等的调节，参与细胞迁移和粘附过程，促进肿瘤细胞转移^[3, 5]^。抑制NF-κB活性可下调MMP-2和MMP-9的表达，肺癌的侵袭性、转移能力受抑^[14]^。本研究中NF-κBp65活性受抑，A549细胞迁移、粘附能力均下降，表明这可能是NF-κBp65活性受抑造成MMPs、VCAM-1、ICAM- 1等的表达下调的效应。

综上所述，NF-κBp65活性受抑可间接影响其抑制因子IκBα蛋白水平，慢病毒介导的shRNA干扰技术可有效的用于以NF-κB为靶点的肿瘤的基因治疗中，通过抑制NF-κB活化，明显降低肿瘤细胞的迁移、粘附能力，为肿瘤基因治疗中将NF-κB作为肿瘤治疗或预防的靶点提供了有利的证据。
